# The impact of polymorphisms in *STAT6* on treatment outcome in HCV infected Taiwanese Chinese

**DOI:** 10.1186/1471-2172-14-21

**Published:** 2013-05-08

**Authors:** Yun-Ping Lim, Yu-An Hsu, Kun-Hsi Tsai, Fuu-Jen Tsai, Cheng-Yuan Peng, Wen-Ling Liao, Dong-Zong Hung, Ni Tien, Chien-Yih Lin, Lei Wan

**Affiliations:** 1 Department of Pharmacy, College of Pharmacy, China Medical University, Taichung 40402, Taiwan; 2 Department of Emergency, Toxicology Center, China Medical University Hospital, Taichung 40447, Taiwan; 3 Department of Life Science, National Tsing Hua University, Hsinchu 30013, Taiwan; 4 Department of Emergency Medicine, Chi Mei Hospital, Liouying, Tainan, Taiwan; 5 School of Chinese Medicine, China Medical University, No. 91, Hsueh-Shih Road, Taichung 40402, Taiwan; 6 Department of Internal Medicine, China Medical University, Taichung 40402, Taiwan; 7 Division of Digestive System and Gastroenterology, Department of Internal Medicine, China Medical University Hospital, Taichung 40447, Taiwan; 8 Center for Personalized Medicine, China Medical University Hospital, Taichung 40447, Taiwan; 9 Department of Laboratory Medicine, China Medical University Hospital, Taichung 40447, Taiwan; 10 Department of Veterinary Medicine, National Chung Hsing University, Taichung 40227, Taiwan; 11 Department of Biotechnology, Asia University, Taichung 41354, Taiwan

**Keywords:** Hepatitis C virus, Standard of care treatment, Sustained virological response, Signal transducer and activator of transcription 6

## Abstract

Genetic polymorphisms observed in various disease states associated with sensitivity or resistance to specific treatments have been a robust area of investigation for decades, with the potential to allow clinicians to make evidence-based decisions on the appropriate course of treatment. This study aimed to evaluate whether genetic polymorphisms of the signal transducer and activator of transcription 6 gene (*STAT6*) could be associated with a sustained virological response (SVR) among patients infected with hepatitis C virus genotypes 1 and 2 (HCV-1 and HCV-2) who were treated with peginterferon plus ribavirin (PEG-IFNα-RBV). We analyzed the associations between SVR to PEG-IFNα-RBV therapy and 4 single nucleotide polymorphisms (SNPs) in *STAT6*. This study included Taiwanese Chinese patients infected with either HCV-1 (n = 265) or HCV-2 (n = 195) in the presence or absence of an SVR. Among the *STAT6* SNPs examined, the dosage effect of the A allele and allele frequency in rs1059513 were inversely correlated with SVR in patients infected with HCV-1 (*P* = 0.0179 and *P* = 0.0235, respectively). This effect was not observed in patients infected with HCV-2. The GG, GGG, and GGGC *STAT6* haplotypes comprising 2, 3, and 4 SNPs (rs1059513, rs703817, rs324015, and rs3024974) were found to be associated with SVR, and their presence may increase the probability of a successful treatment outcome in patients infected with HCV-1 (*P* = 0.0273, 0.0352, and 0.0368, respectively). Moreover, a multivariate logistic regression model for predicting an SVR revealed that the presence of the GGGC haplotype carriers mutually affected the outcome of PEG-IFNα-RBV treatment. The presence of *STAT6* SNPs and the association with SVR demonstrated that *STAT6* polymorphisms might influence the therapeutic outcomes of patients infected with HCV-1 under standard-of-care (SOC) treatment.

## Background

Hepatitis C is a common infectious disease caused by hepatitis C virus (HCV). Infection with HCV is the most common cause of chronic liver disease, hepatoma, and liver transplantation [1-3]. Typically, HCV spreads through exposure to infected blood, especially via intravenous drug abuse, which is a major route of transmission. In addition to blood-borne exposure, HCV may also spread through sexual contact and vertical transmission, albeit at lower risk [3]. It is estimated that approximately 170 million people worldwide are infected with HCV [4,5].

The standard-of-care (SOC) treatment for chronic hepatitis C includes a combination of pegylated interferon alpha (PEG-IFNα) and ribavirin (RBV) [6,7]. The effectiveness of 2 types of peginterferon, alfa-2a and alfa-2b, both differing in their pharmacokinetic and chemical properties, has been determined [8,9]. Peginterferon alfa-2a plus ribavirin is more effective in treating chronic hepatitis C than interferon alfa-2b plus ribavirin or peginterferon alfa-2a alone [8]. In general, successful treatment is defined as the persistent absence of serum HCV RNA for 6 months or longer post therapy, known as a sustained virological response (SVR) [10].

IFNα exert multifunctional biological activities and consists of highly homologous proteins mediated by binding to the receptor chains, IFNαR1 or IFNαR2 [11]. Binding of IFNα to its receptor leads to its activation via two receptor-associated tyrosine kinases (Tyk2) and Janus kinases 1 and 2 (JAK kinases 1 and 2), which in turn triggers the activation of cytoplasmic factors, namely, the STAT (signal transducers and activators of transcription) family of proteins [11]. STAT6 is a cytosolic protein, the tyrosine phosphorylation of which is catalyzed by JAK kinases 1 and 2 [11]. Subsequently, phosphorylated STAT6 proteins dimerize via their SH2 domains and translocate into the nucleus, where they bind to response elements of target genes and regulate cytokine production [11,12]. STAT6 induces two forms of cytokines, IL-4 and IL-13. Formation of STAT homo/heterodimer in response to IFNα activation may initiate transcription of several genes, including genes encoding the transcription factor IFN regulatory factor 1 (IRF-1) and IFN-stimulated gene factor 3 (ISGF3). The ISGF3 complex may trigger several subsets of genes termed IFN-stimulated response elements (ISREs) by binding to different parts of *cis*-acting elements [12]. The JAK/STAT signaling pathway is activated when the cytokine interleukins 4 (IL-4), IL-12, and/or type I interferon (IFN) bind to their respective receptors. Since STAT6 protein is essential for responsiveness to IL-4 and IL-12, *STAT6*-deficient mice do not produce significant numbers of type 2 CD4^+^ T cells, thereby blocking the differentiation of these cells [13,14]. Furthermore, multiple studies have demonstrated that *STAT6* knockout mice are resistant to tumor growth and spontaneous metastatic disease [15]. Therefore, IFN-mediated activation of STAT6 occurs via production of secreted IL-1Ra during inflammatory responses [16]. STAT6 activation has been reported to play an important role in the regulation of genes responsible for their antitumor or antiviral effects.

Previous studies have demonstrated that the SVR rates for patients infected with HCV genotype 1 (HCV-1) are significantly lower than the rates for those who are infected with HCV genotype 2 (HCV-2). In order to validate the potentially useful prognostic biomarkers that are able to predict SVR during early treatment, previous studies have used real-time PCR to estimate the expression profiles of 68 messenger RNAs isolated from HCV-1 infected patients, as well as their correlation with SVR [17]. Investigators found that *STAT6* expression levels are highly correlated with SVR. It has been suggested that, in addition to inducing important molecular pathways, the expression of several early genes during HCV therapy may also influence the capacity of residual SVR.

In the present study, we investigated the association between single nucleotide polymorphisms (SNPs) in the *STAT6* gene and their susceptibility to SVR in Chinese patients in Taiwan receiving PEG-IFNα-RBV treatment. Our results support the hypothesis that *STAT6* is a potential candidate gene for predicting therapeutic outcomes of HCV-1 infected patients.

## Methods

### Patients

In the present study, 265 patients infected with HCV-1 and 195 patients infected with HCV-2 at the China Medical University Hospital, Taichung, Taiwan were enrolled and actively observed. All participating subjects provided informed consent, and the study protocol was approved by the chairman of the Ethics Committee of the China Medical University Hospital in accordance with the guidelines of the Declaration of Helsinki. Diagnosis of HCV infection was based on persistent elevation of serum transaminase levels for at least 6 months and serum anti-HCV positivity, coupled with the periodic detection of serum HCV RNA. Patients positive for hepatitis B surface antigen, antibodies, and human immunodeficiency virus 1 and 2 were excluded in this study. Patients received weekly injections of PEG-IFNα (1.5 μg/kg body weight) plus body weight–adapted doses of RBV (800 mg/day for < 60 kg; 1000 mg/day for 60–75 kg; 1200 mg/day for > 75 kg) by oral administration for 48 weeks (HCV-1) or 24 weeks (HCV-2).

### SNP selection

Selection of representative *STAT6* SNPs was based on SNP genotype information, downloaded in December 2008 from the HapMap Chinese Han in Beijing (CHB) + JPT population. HapMap genotypes were further analyzed via Haploview software (version 4.2; Broad Institute, Cambridge, MA, USA). Tag SNPs were selected by using the Tagger function according to the following criteria: (1) a minor allele frequency (MAF) in the HapMap CHB + JPT population of > 0.10; and (2) a genotyping score of ≥ 0.6 (Illumina, Inc., San Diego, CA), as recommended by the manufacturer, to achieve a successful genotyping rate. Four SNPs in the *STAT6* gene met the above criteria and were selected, including rs1059513 (S1; A/G at 3′ UTR), rs703817 (S2; A/G at 3′ UTR), rs324015 (S3; A/G at 3′ UTR), and rs3024974 (S4; C/T at boundary of intron 17).

### HCV genotyping and RNA measurements

Genotyping of HCV according to the classification of Simmonds *et al.*[18] was performed by a reverse hybridization assay (INNO LiPA HCV-II; Innogenetics, Ghent, Belgium). The virological response was assessed by qualitative HCV RNA assays with a lower sensitivity of 30–50 IU/ml (HCV Amplicor™ 2.0, Roche Diagnostics, Branchburg, NJ, USA). According to the qualitative HCV RNA results (reported in copies/mL), patients were defined as (1) sustained virological responders (HCV RNA undetectable at week 24 posttreatment) and (2) non-sustained virological responder (HCV RNA detected at week 24 posttreatment). Thus, the subjects were classified as either SVR (+) or SVR (−), respectively.

### Genomic DNA extraction and genotyping

Genomic DNA was isolated from the peripheral blood of all participants by using a commercial kit (Genomic DNA kit; QIAGEN, Valencia, CA, USA) according to the manufacturer’s instructions. Four *STAT6* polymorphisms were detected by an allele-specific extension method and ligation assay kit (Illumina, San Diego, CA, USA), in accordance with the manufacturer’s instructions.

### Statistical analysis

Gender, age, body mass index (BMI), and viral load differences between SVR (+) and SVR (−) groups were estimated using the Mann–Whitney *U* test. The differences between genotypes and each of the above mentioned parameters were estimated using the Kruskal–Wallis test. The association of each SNP with SVR was assessed using the chi-square (*χ*^2^) test or Fisher’s exact test. Genotype and allele frequencies in SVR (+) and SVR (−) patients were compared, and odds ratios (ORs) with a 95% confidence interval (CI) were estimated by applying an unconditional logistic regression model. Haplotype analysis using a sliding window mode and Hardy–Weinberg equilibrium (HWE) analysis were performed using the PLINK program (version 1.07; http://pngu.mgh.harvard.edu/purcell/plink/) [19]. The distribution of each haplotype frequency was identified using Phase version 2.1 (University of Chicago, Chicago, IL, USA), a computational tool based on Bayesian methods [20]. Multivariate logistic regression analysis was used to predict the independent variables that correlated with a therapeutic response. An OR at a 95% CI was employed for this analysis. Statistical tests were performed using SPSS software (version 20.0 for Windows; SPSS Inc., Chicago, IL, USA). Statistical significance was defined as *P* < 0.05 and was two-sided. Screening and construction of linkage disequilibrium (LD) plots were performed using Haploview software (version 4.2).

## Results

### Patient characteristics

In this study, 460 patients infected with HCV-1 (n = 265) and HCV-2 (n = 195) were successfully enrolled. Patients infected with HCV-2 achieved SVR at higher rates than patients infected with HCV-1 (91.3% vs. 61.5%). The basic characteristics and clinical information of all patients infected with HCV-1 and HCV-2 are displayed in Table 1. The information is further stratified by HCV genotype. We observed several significant differences between SVR (+) and SVR (−) groups, including their age (starting with date of entry), body mass index (BMI), platelets and viral load in HCV-1–infected patients (*P* = 0.002, *P* = 0.026, *P* = 0.002, and *P* = 0.001, respectively). Some effects were not observed significantly in HCV-2 infected populations. However, statistically significant differences in the aforementioned variables were not observed among the 4 SNPs.

**Table 1:** Characteristics of the HCV genotype 1 and 2 infected patients receiving PEG-IFNα-RBV therapy

	**HCV genotype 1 (HCV-1)**				**HCV genotype 2 (HCV-2)**			
	**All**	**SVR (+)**	**SVR (−)**	***P *value**	**All**	**SVR (+)**	**SVR (−)**	***P *value**
**Number of patients**	265	163	102	-	195	178	17	-
**Gender (males/females)**	129/136	84/79	45/57	0.241	88/107	81/97	7/10	0.732
**Age (mean ± SD)**	52.17 ± 10.27	50.69 ± 10.60	54.52 ± 9.29	0.002^a^	51.62 ± 10.89	51.15 ± 11.16	56.47 ± 5.86	0.050^a^
**BMI (mean ± SD)**	24.6 ± 3.1	24.2 ± 2.9	25.2 ± 3.3	0.026^a^	24.5 ± 3.5	24.5 ± 3.6	24.5 ± 2.7	0.863^a^
**Degree of inflammatory activity (A0/A1-3)**^**c**^	35/230	17/146	18/84	0.0913^b^	22/173	18/160	4/13	0.0948^b^
**Stage of fibrosis (F0/F1-4)**^**c**^	16/249	6/157	10/92	0.0417^b^	10/185	7/171	3/14	0.0143^b^
**AST (U/L) (mean ± SD)**		76.15 ± 41.24	90.91 ± 61.12	0.049^a^		84.26 ± 63.45	78.12 ± 34.03	0.744^a^
**ALT (U/L) (mean ± SD)**		118.56 ± 75.99	108.05 ± 74.97	0.328^a^		120.67 ± 104.63	100.71 ± 44.76	0.822^a^
**Platelet (× 10**^**3**^**/μl) (mean ± SD)**		176.4 ± 56.7	154.9 ± 59.5	0.002^a^		174.4 ± 52.4	125.5 ± 41.8	0.001^a^
**Viral load (× 10**^**6**^**)**	12.1 ± 16.4	11.0 ± 16.5	13.9 ± 16.2	0.001^a^	11.0 ± 19.0	10.3 ± 17.8	18.4 ± 27.9	0.334^a^

Abbreviations: *SVR* sustained virological response, *BMI* body mass index.

^a^ Calculated by Mann–Whitney *U* test.

^b^ Calculated by the *χ*^2^ test.

^c^ The inflammatory activity and the stage of fibrosis were graded according to the Metavir scoring system [21].

The chromosome position, Hardy–Weinberg equilibrium (HWE), and MAF in individuals bearing the aforementioned 4 *STAT6* SNPs genotypes examined in this study are displayed in Table 2. The SNPs were in accordance with HWE (*P* > 0.05).

**Table 2:** **Four single nucleotide polymorphisms in the *STAT6 *gene in 265 HCV-1 and 195 HCV-2 infected patients receiving PEG-IFNα-RBV therapy with or without a SVR in a Chinese population in Taiwan**

**SNPs**	**Position in *STAT6***	**Chromosome position**^**a**^	**Alleles (1/2)**	**HCV-1**			**HCV-2**		
				**HWE (*P *value)**	**MAF**		**HWE (*P *value)**	**MAF**	
					**SVR (+)**	**SVR (−)**		**SVR (+)**	**SVR (−)**
rs1059513 (S1)	3′ UTR	57489709	A/G	0.3808	0.0982	0.0441	1	0.0646	0.0589
rs703817 (S2)	3′ UTR	57489828	A/G	0.7565	0.2454	0.3119	0.7054	0.2612	0.1471
rs324015 (S3)	3′ UTR	57490100	A/G	0.5347	0.4448	0.4406	0.5655	0.4831	0.4118
rs3024974 (S4)	Intron 17 (boundary)	57492745	C/T	0.8524	0.2086	0.2108	0.6503	0.1949	0.2059

Abbreviations: *SNP* single nucleotide polymorphism, *HWE* Hardy-Weinberg equilibrium, *MAF* minor allele frequency.

^a^ Chromosome positions refer to the sequence in the NCBI database (built 37.3).

### Association of tagging SNPs of *STAT6* with therapeutic response and SVR

The genotype frequencies and allelic dose-dependent association of each SNP with responsiveness to PEG-IFNα-RBV therapy are displayed in Table 3. In genotype association tests, only genotypes from rs1059513 were significantly associated with SVR in HCV-1 infected patients (*P* = 0.0179). When the A/A and A/G genotypes were compared, a decreased crude OR of 0.40 (95% CI, 0.18–0.87; *P* = 0.0179) for SVR (+) versus SVR (−) groups was observed. However, no genotype was significantly associated with SVR in the HCV-2 infected population.

**Table 3:** **Genotype frequencies of *STAT6 *single nucleotide polymorphisms (SNPs) in HCV-1 and HCV-2 infected patients receiving PEG-IFNα-RBV therapy with and without a SVR in a Chinese population in Taiwan**

**HCV-1**					**HCV-2**				
**SNP ID**	**SVR (+)**	**SVR (−)**	***P***	**OR (95% CI)**	**SNP ID**	**SVR (+)**	**SVR (−)**	***P***	**OR (95% CI)**
	**N (%)**	**N (%)**				**N (%)**	**N (%)**		
**rs1059513**					**rs1059513**				
A/A	131 (80.4)	93 (91.2)		0.40 (0.18, 0.87)	A/A	155 (87.1)	15 (88.2)		0.90 (0.19, 4.19)
A/G	32 (19.6)	9 (8.8)	0.0179*	1	A/G	23 (12.9)	2 (11.8)	0.8916	1
G/G	0 (0.0)	0 (0.0)	-	-	G/G	0 (0.0)	0 (0.0)	-	-
A/A + A/G	163 (100.0)	102 (100.0)	-	-	A/A + A/G	178 (100.0)	17 (100.0)	-	-
**rs703817**^**a**^					**rs703817**				
A/A	11 (6.7)	9 (8.9)		0.61 (0.24, 1.58)	A/A	11 (6.2)	0 (0.0)		-
A/G	58 (35.6)	45 (44.6)		0.64 (0.38, 1.09)	A/G	71 (39.9)	5 (29.4)		1.78 (0.60, 5.27)
G/G	94 (57.7)	47 (46.5)	0.2103	1	G/G	96 (53.9)	12 (70.6)	0.3223	1
A/A + A/G	69 (42.3)	54 (53.5)	0.0780	0.64 (0.39, 1.05)	A/A + A/G	82 (46.1)	5 (29.4)	0.1869	2.05 (0.69, 6.06)
**rs324015**^**a**^					**rs324015**				
A/A	34 (20.9)	20 (19.8)		1.05 (0.52, 2.12)	A/A	39 (21.9)	6 (35.3)		0.43 (0.10, 1.85)
A/G	77 (47.2)	49 (48.5)		0.97 (0.55, 1.71)	A/G	94 (52.8)	8 (47.1)		0.78 (0.20, 3.09)
G/G	52 (31.9)	32 (31.7)	0.9723	1	G/G	45 (25.3)	3 (17.6)	0.4339	1
A/A + A/G	111 (68.1)	69 (68.3)	0.9704	0.99 (0.58, 1.69)	A/A + A/G	133 (74.7)	14 (82.4)	0.4851	0.63 (0.17, 2.31)
**rs3024974**					**rs3024974**^**b**^				
C/C	105 (64.4)	61 (59.8)		0.34 (0.07, 1.62)	C/C	113 (63.9)	11 (64.7)		2.05 (0.22, 19.19)
C/T	48 (29.5)	39 (38.2)		0.25 (0.05, 1.19)	C/T	59 (33.3)	5 (29.4)		2.36 (0.23, 24.33)
T/T	10 (6.1)	2 (2.0)	0.1285	1	T/T	5 (2.8)	1 (5.9)	0.7623	1
C/C + C/T	153 (93.9)	100 (98.0)	0.1118	0.31 (0.07, 1.43)	C/C + C/T	172 (97.2)	16 (94.1)	0.4867	2.15 (0.24, 19.55)

^a^ Contains 1 missing data point in the SVR (−) group.

^b^ Contains 1 missing data point in the SVR (+) group.

Abbreviations: *SNP* single nucleotide polymorphism, *SVR* sustained virological response, *OR* odds ratio, *CI* Confidence interval.

Genotype frequencies were determined by *χ*^2^ test using 2 × 3 or 2 × 2 tables as appropriate. Odds ratios and 95% CI per genotype were estimated by unconditional logistic regression. *P* values less than 0.05 were considered statistically significant, and are denoted with an asterisk.

The distributions of the aforementioned 4 *STAT6* SNPs allele frequencies between SVR (+) and SVR (−) groups in patients infected with HCV-1 and HCV-2 are displayed in Table 4. Compared with SVR (+) individuals, patients harboring the A allele of SNP rs1059513 were at an increased risk for non-responsiveness to SOC treatment (*P* = 0.0235; OR = 0.42, 95% CI, 0.20–0.91), with a significance observed exclusively in patients infected with HCV-1. Thus, HCV-1 infected individuals with *STAT6* rs1059513 A/A genotype and carrying the A allele may be at increased risk for non-responsiveness to PEG-IFNα-RBV treatment.

**Table 4:** **Allele frequencies of *STAT6 *single nucleotide polymorphisms in HCV-1 and HCV-2 infected patients receiving PEG-IFNα-RBV therapy with and without a SVR in a Chinese population in Taiwan**

**HCV-1**					**HCV-2**				
**SNP ID**	**SVR (+)**	**SVR (−)**	***P***	**OR (95% CI)**	**SNP ID**	**SVR (+)**	**SVR (−)**	***P***	**OR (95% CI)**
	**N (%)**	**N (%)**				**N (%)**	**N (%)**		
**rs1059513**					**rs1059513**				
A allele	294 (90.2)	195 (95.6)		0.42 (0.20, 0.91)	A allele	333 (93.5)	32 (94.1)		0.90 (0.20, 4.01)
G allele	32 (9.8)	9 (4.4)	0.0235*	1	G allele	23 (6.5)	2 (5.9)	0.8954	1
**rs703817**^**a**^					**rs703817**				
A allele	80 (24.5)	63 (31.2)		0.72 (0.49, 1.06)	A allele	93 (26.1)	5 (14.7)		2.05 (0.77, 5.45)
G allele	246 (75.5)	139 (68.8)	0.0948	1	G allele	263 (73.9)	29 (85.3)	0.1425	1
**rs324015**^**a**^					**rs324015**				
A allele	145 (44.5)	89 (44.1)		1.02 (0.71, 1.45)	A allele	172 (48.3)	20 (58.8)		0.65 (0.32, 1.34)
G allele	181 (55.5)	113 (55.9)	0.9249	1	G allele	184 (51.7)	14 (41.2)	0.2416	1
**rs3024974**					**rs3024974**^**b**^				
C allele	258 (79.1)	161 (78.9)		1.01 (0.66, 1.56)	C allele	285 (80.5)	27 (79.4)		1.07 (0.45, 2.56)
T allele	68 (20.9)	43 (21.1)	0.9518	1	T allele	69 (19.5)	7 (20.6)	0.8777	1

^a^ Contains 1 missing data point in the SVR (−) group.

^b^ Contains 1 missing data point in the SVR (+) group.

Abbreviations: *SNP* single nucleotide polymorphism, *SVR* sustained virological response, *OR* odds ratio, *CI* confidence interval.

Allele frequencies were determined by *χ*^2^ test using 2 × 2 tables. Odds ratios and 95% CI per allele were estimated by unconditional logistic regression. *P* values less than 0.05 were considered statistically significant, and are denoted with an asterisk.

### Frequencies of the *STAT6* haplotypes and the association with SVR

We performed a comparative analysis of haplotype frequencies and treatment responses by adjustment for gender as a covariate by using the PLINK program. The results of the overall global test and haplotype frequencies are displayed in Table 5. The analysis was introduced by the sliding window mode to examine the potential sizes of all haplotypes (number SNPs per haplotype). As a result, there were 10 sliding windows, 1 of which (the omnibus test) was significantly associated with SVR in HCV-1 infected patients (*P* < 0.05).

**Table 5:** Details of sex-adjusted haplotype frequency analysis for 2-SNP, 3-SNP, and 4-SNP windows showing the most significant results among all possible sliding windows in HCV-1 infected patients

**Haplotypes**	**SVR (+)**	**SVR (−)**	**OR**	***P *value**
rs1059513-rs703817 (S1-S2)				
OMNIBUS	-	-	-	0.0401*
GG (22)	0.0982	0.0450	2.31	0.0273*
rs1059513-rs703817-rs324015 (S1-S2-S3)				
OMNIBUS	-	-	-	0.1101
GGG (222)	0.0948	0.0447	2.24	0.0352*
rs1059513-rs703817-rs324015-rs3024974 (S1-S2-S3-S4)				
OMNIBUS	-	-	-	0.1894
GGGC (2221)	0.0960	0.0457	2.22	0.0368*

Abbreviations: *SVR* sustained virological response, *OR* odds ratio.

*P* values less than 0.05 were considered statistically significant, and are denoted with an asterisk.

In HCV-1 infected populations possessing haplotype GG, the window S1-S2 (composed of rs1059513 and rs703817), gave the most significant *P* value (*P* = 0.0273; OR = 2.31). Haplotype-specific analyses showed that the GGG haplotype (S1-S2-S3, composed of rs1059513, rs703817, and rs324015) may increase the rate of SVR (*P* = 0.0352; OR = 2.24) in the SVR (+) groups compared with the SVR (−) groups. The window S1-S2-S3-S4 with the GGGC haplotype (composed of rs1059513, rs703817, rs324015, and rs3024974) was significantly associated with a higher rate of SVR in HCV-1 infected patients (OR = 2.22; *P* = 0.0368). Furthermore, the results demonstrated that HCV-1 infected patients with therapeutic response bearing the haplotypes GG, GGG, and GGGC, appeared more frequently in SVR (+) groups than in SVR (−) groups. However, the haplotypes did not play a significant role in HCV-2 infected patients (data not shown). Thus, in the HCV-1 infected individuals, haplotype-specific analyses showed that the aforementioned haplotypes are associated with an increase success rate of SVR and may play a role in the therapeutic outcomes of PEG-IFNα-RBV treatment in HCV-1 infected patients. Table 6 shows that 9.5% of 163 SVR (+) HCV-1 infected patients possessed the GGGC haplotype. The GGGC haplotype carriers achieved a significantly higher SVR rate compared with non-GGGC carriers (95% CI, 1.06–4.89, OR = 2.28; *P* = 0.0306). These findings confirmed the results of our analyses of genotypes, allele frequencies, and haplotypes.

**Table 6:** **Distribution of haplotype frequencies from SNPs of *STAT6 *gene in 265 HCV-1 infected populations and their associations with SVR**

Haplotypes^*^	**SVR (+) (%)**	**SVR (−) (%)**	***P *Global**^**†**^	***P *individual**^**§**^	**OR (95% CI)**
AGGT	66 (20.3)	40 (19.6)	0.2126	0.8583	1.04 (0.67, 1.61)
AGGC	4 (1.2)	2 (1.0)		0.7940	1.25 (0.23, 6.91)
AGAT	2 (0.6)	3 (1.5)		0.3206	0.41 (0.07, 2.50)
AGAC	144 (44.2)	87 (42.6)		0.7305	1.06 (0.75, 1.51)
AAGC	78 (23.9)	63 (30.9)		0.0778	0.70 (0.48, 1.04)
GGGC	31 (9.5)	9 (4.4)		0.0306*	2.28 (1.06, 4.89)
GGAC	1 (0.3)	0 (0.0)		0.4285	-

^*^Order of SNPs comprising the *STAT6* haplotypes: rs1059513, rs703817, rs324015 and rs3024974. The haplotypes were identified by the Bayesian statistical method available in the program Phase 2.1.

^**†**^ Global test for haplotypes frequency in relation to SVR was determined by *χ*^2^ test using 2 × 7 contingency tables.

^**§**^ Individual haplotype frequency in relation to SVR was determined by *χ*^2^ test using 2 × 2 contingency tables. *P* values less than 0.05 were considered statistically significant, and are denoted with an asterisk. ORs and 95% CI were estimated by unconditional logistic regression model.

**Figure 1:**
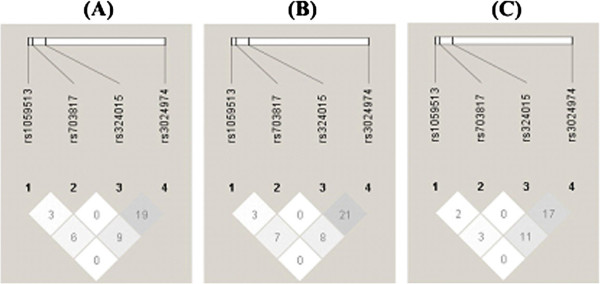
**Linkage disequilibrium plot of the *STAT6 *gene for the analyzed SNPs.** The figure shows the output of Haploview (version 4.2) linkage disequilibrium plot where each square (with r^2^ values written within the box correspond to r^2^ values × 100 as linkage disequilibrium measure range) represents a pairwise linkage disequilibrium relationship between the two SNPs. Darkest colored squares indicate high linkage disequilibrium (r^2^ = 1); mid colored squares indicate r^2^ values between 0 and 1; lightest colored squares indicate low linkage disequilibrium (r^2^ = 0). Figure depicts the linkage disequilibrium pattern in (**A**) all HCV-1 infected patients; (**B**) HCV-1 infected PEG-IFNα-RBV therapy with SVR (+) patients; (**C**) HCV-1 infected PEG-IFNα-RBV therapy with SVR (−) patients.

We performed LD analysis, and the results were viewed using Haploview software (version 4.2). Our results indicated that a low degree of pairwise LD among these SNPs in all HCV-1 infected populations in the presence or absence of SVR. The graphical summary of LD (r^2^ values) among tested 4 *STAT6* SNPs at different loci is displayed in Figure 1. Four tag SNPs were selected and designated in sequential order.

### Multivariate logistic regression analysis

We subsequently determined whether independent factors affected the outcome of PEG-IFNα-RBV therapy. To this end, we performed multivariate logistic regression analyses with respect to GGGC/non-GGGC haplotypes, age, BMI, and HCV RNA levels (low/high). This analysis revealed that the GGGC haplotype carrier acts as an independent predictor of the virological response outcome of PEG-IFNα-RBV therapy (*P* = 0.043; OR = 2.32, 95% CI, 1.03–5.23) (Table 7). Furthermore, age and HCV viral load may also act together to influence the outcome of PEG-IFNα-RBV treatment (*P* = 0.012; OR = 0.97, 95% CI, 0.94–1.00; and *P* = 0.050; OR = 8.11, 95% CI, 0.98–67.32, respectively).

**Table 7:** Predictive factors associated independently with the SVR to PEG-IFNα-RBV therapy in HCV-1 infected populations by multivariate logistic regression analysis

**Parameter**	***P *value**	**OR (95% CI)**
Haplotype^§^ (GGGC/non-GGGC)	0.043*	2.32 (1.03, 5.23)
Age	0.012*	0.97 (0.94, 1.00)
BMI	0.060	0.92 (0.85, 1.00)
HCV-RNA level^δ^ (low/high)	0.050*	8.11 (0.98, 67.32)

Abbreviation: *CI* confidence interval, *BMI* body mass index.

^§^ The order of the SNPs comprising the *STAT6* haplotype was rs1059513, rs703817, rs324015, and rs3024974.

^δ^ Low HCV-RNA level: <100 KIU/ml by Amplicore-monitor assay. Odds ratios (OR) of having a SVR to PEG-IFNα-RBV therapy were calculated. *P* values less than 0.05 were considered statistically significant, and are denoted with an asterisk.

## Discussion

The present study examined the genetic association of HCV infection under PEG-IFNα-RBV therapy on a responsive gene, *STAT6*, and responsiveness of treatment. We confirmed that this gene, located on chromosome 12, is a novel candidate susceptibility gene. The results showed that rs1059513 at the 3′ UTR of *STAT6* may be associated with SVR rates under SOC treatment. Therapeutic outcome association studies showed that the presence of the GGGC haplotype was significantly associated with a higher rate of SVR (OR = 1.06–4.89) in HCV-1 infected but not HCV-2 infected populations. By using multivariate logistic regression analyses, we observed that the GGGC haplotype, age of entry, and viral load may act interactively to influence the therapeutic outcomes. This result suggests that the SVR rates of PEG-IFNα-RBV treatment could, at least partially, depend on STAT6 activation.

Thus far, the standard treatment regimen for chronic hepatitis C infection has been a combination of PEG-IFNα and RBV [8]. About 50% of treatment-naïve populations will typically achieve SVR by using this regimen [8]. The SVR rate is markedly higher in HCV-2/3 infected patients than in HCV-1 infected patients (~ 85% versus 40%) [8]. In order to identify the biomarkers that distinguish the profiles of HCV therapeutic responsiveness, Younossi and colleagues [17] collected RNA transcripts from peripheral blood mononuclear cells (PBMCs) of 68 HCV-infected patients under PEG-IFNα-RBV treatment. After preparing expression profiles of patients under this treatment, the investigators observed that genetic expression of STAT6 and cytokine signaling 1 are putative predictive markers that correlate with SVR [17]. In support of this finding, we also observed a correlation between *STAT6* genetic polymorphisms and SVR.

During viral infections, innate immunity remains the most important first line of defense in the body. Viral nucleic acids are detected by endosomal Toll-like receptors (TLRs) and cytoplasmic RIG-I–like receptors (RLRs), which subsequently induce the production of type 1 IFN to inhibit viral replication [22]. Consequently, many cytokines (including type 1 IFNs) trigger the IFN-stimulated gene expression cascade through the canonical JAK-STAT signaling pathway [23]. Previous studies have shown that STAT6 activation is ubiquitously detected during viral infections, concluding that STAT6 may play a fundamental role in the defense mechanism against viral infections [24]. Viral load, age of entry into the study, and BMI were not significantly different among the *STAT6* genotypes of our enrolled populations. Thus, it is unlikely that specific *STAT6* genotypes predispose individuals to infection with HCV or contribute to the process of spontaneous viral elimination.

Studies using a microarray platform to examine the differential gene expression profiles of IL-4–stimulated B cells between wild-type and *Stat6*−/− mice were recently performed [25]. Seventy known genes were found to be differentially expressed with significant discrepancies between the 2 mouse groups, which were all negatively or positively regulated by *STAT6* via a palindromic consensus sequence TTC(N)_2-4_GAA [26]. In addition, STAT6 remains a critical determinant in the polarization and differentiation process of T-helper type 2 (Th2) cells in a *Stat6* knockout mouse model [27]. The expression levels of Th2-related cytokines (including IL-4, IL-5, and IL-13) were diminished in this mouse model [27]. Moreover, STAT6 may trigger a Th1 response by enhancing the production of IL-12 through the inhibition of IL-10 production in dendritic cells [28]. Therefore, STAT6 is also a critical factor in the homeostasis of inflammatory and hypersensitivity immune responses other than those associated with viral immunity.

Several reports have provided strong evidence that patients infected with HCV-1 will have approximately a 50% probability (in Caucasians) or 80% probability (in African Americans) of a poor response toward PEG-IFNα-RBV treatment [8]. Although viral clearance rates have been strongly associated with clinical features of HCV, for example: gender, age < 40 years, low HCV RNA levels pre-treatment, in the absence of liver cirrhosis, and HCV genotypes 2/3 [29]. Table 7 lists 11 patients with low viremia. Fried *et al.*[8] concluded that HCV RNA level is one of the main factors affecting treatment outcome; therefore, we performed multivariate analysis to check whether HCV RNA level is an independent factor of treatment outcome. We first determined the HCV RNA baseline without categorizing the patients; however, in this analysis, *P* = 0.182 and OR = 1.00. This result led us to conclude that it is not the best model for prediction of treatment outcome. Therefore, we categorized the HCV RNA level according to the description of Fujimoto *et al.*[30], with low HCV RNA level <100 KIU/ml by Amplicore-monitor assay and performed statistical analysis, and the results for this analysis are more obviously (*P* = 0.050, OR = 8.11).

Many investigators continue to monitor the host genetic factors that may relate to clinical outcomes to provide custom-made therapy for HCV infection. In our study, the overall achievement response rates of SVR were less than 62% and 92% in the HCV-1 and HCV-2 populations, respectively. Thus, a reliable prediction of a subject as a potential non-viral responder (NVR) at the start of treatment would allow the clinician to make appropriate, evidence-based decisions on the compatibility of future treatment, thereby reducing side effects and/or the cost of treatment.

The contributions of host genetic factors to HCV-2/3 clearance are relatively small compared with HCV-1 clearance, because the former is more likely to be eliminated by SOC therapy [8]. In the present study, we observed an association between the *STAT6* haplotype GGGC and the rate of SVR, although its statistical significance in the HCV-1 infected populations based on estimation by multivariate logistic regression was marginal. The frequency of the GGGC allele in our population was small, whether the frequency of this haplotype is significant in other ethnic groups remains unknown. We propose that this STAT6 haplotype GGGC might play a role in the outcome of PEG-IFNα-RBV therapy (adjusted r^2^ = 11.2%). The linkage among the *STAT6* polymorphisms and SVR should be confirmed in future studies with larger sample sizes. Moreover, the significance of the genetic effects of *STAT6* on other racial and ethnic groups remains to be elucidated.

The mechanisms of action of the aforementioned 4 SNPs located within the 3′ UTR and intronic regions of *STAT6* gene are hampered by the lack of published functional studies on these polymorphisms and warrants further investigation at the molecular level.

## Conclusion

This study provides evidence that the *STAT6* genetic polymorphisms are related to the susceptibility of HCV-infected patients, especially HCV-1 infected patients to SVR. Furthermore, an outcome measure of haplotype GGGC in the clinical response was identified. Thus, *STAT6* may play an important role in a broad range of antiviral activities under PEG-IFNα-RBV treatment.

## Competing interests

The authors declare that they have no competing interests.

## Authors’ contributions

YPL and LW designed and carried out most of the study. YPL and YAH wrote the manuscript and YPL performed data analysis. KHT, CYP, CYC, WLL, DZH and NT participated in clinical data and information collection. FJT, CYL, and LW conceived and supervised the project and reviewed the manuscript. All authors contributed to and approved the final manuscript by providing constructive suggestions.
